# Stent graft placement for internal iliac artery aneurysm after recanalization of the occluded celiacomesenteric trunk: a case report

**DOI:** 10.1186/s42155-026-00661-3

**Published:** 2026-03-18

**Authors:** Masaki Imaeda, Yasuyuki Onishi, Taro Nakatsu, Takanori Taniguchi

**Affiliations:** 1 Department of Radiology, Tenri Hospital, Tenri, Nara Japan; 2 Department of Diagnostic Imaging and Nuclear Medicine, Kyoto University, Kyoto, Japan; 3 Department of Cardiovascular Surgery, Tenri Hospital, Tenri, Nara Japan

**Keywords:** Internal iliac artery aneurysm, Celiacomesenteric trunk, Stent graft, Collateral pathways, Endovascular treatment, Case report

## Abstract

A 74-year-old male patient with a right internal iliac artery aneurysm had an occluded celiacomesenteric trunk with collateral blood flow via the dilated right middle rectal, superior rectal, and left colic arteries. To prevent hepatic and gastrointestinal ischemia after stent graft placement, the celiacomesenteric trunk was recanalized using angioplasty and stenting. One month later, CT confirmed stent patency and reduced collateral vessel diameter. Two months after recanalization, the right internal iliac artery branches were embolized, and a stent graft was placed from the common to the external iliac artery. One year after the procedure, the patient showed no signs of ischemia, aneurysm enlargement, or endoleaks. In conclusion, a two-stage endovascular approach appears to be effective in preventing ischemic complications while addressing the aneurysm.

**Level of evidence** Level 4, Case Report.

## Introduction

The standard treatment for internal iliac artery (IIA) aneurysms involves embolization of the distal IIA followed by stent graft placement from the common to the external iliac artery [1]. Although effective, this approach may cause buttock claudication, erectile dysfunction, or rare severe ischemia, such as colonic ischemia or spinal cord infarction [2]. The risk of severe ischemic complications may increase when the pelvic arteries, including the IIA, serve as crucial collateral pathways for splanchnic hypoperfusion. The celiacomesenteric trunk (CMT), a vascular variant in which the celiac artery (CA) and superior mesenteric artery (SMA) share a common origin, occurs in approximately 3.4% of individuals [3]. When the CMT is occluded, collateral pathways via the inferior mesenteric artery (IMA) and pelvic arteries, such as the middle rectal artery (MRA), become critical for maintaining blood flow to the liver and gastrointestinal tract [4]. This case report describes an older adult with an enlarging right IIA aneurysm, complicated by CMT occlusion and IMA stenosis, requiring a carefully planned endovascular strategy to prevent intestinal ischemia while effectively addressing the aneurysm.

## Case report

**Fig. 1:**
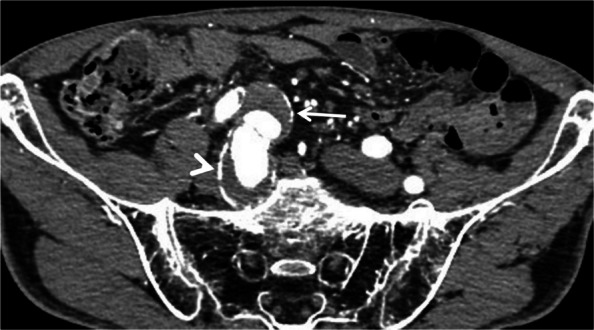
Axial preoperative contrast-enhanced CT. Right common iliac artery aneurysm (arrow) and internal iliac artery aneurysm (arrowhead) are observed

**Fig. 2:**
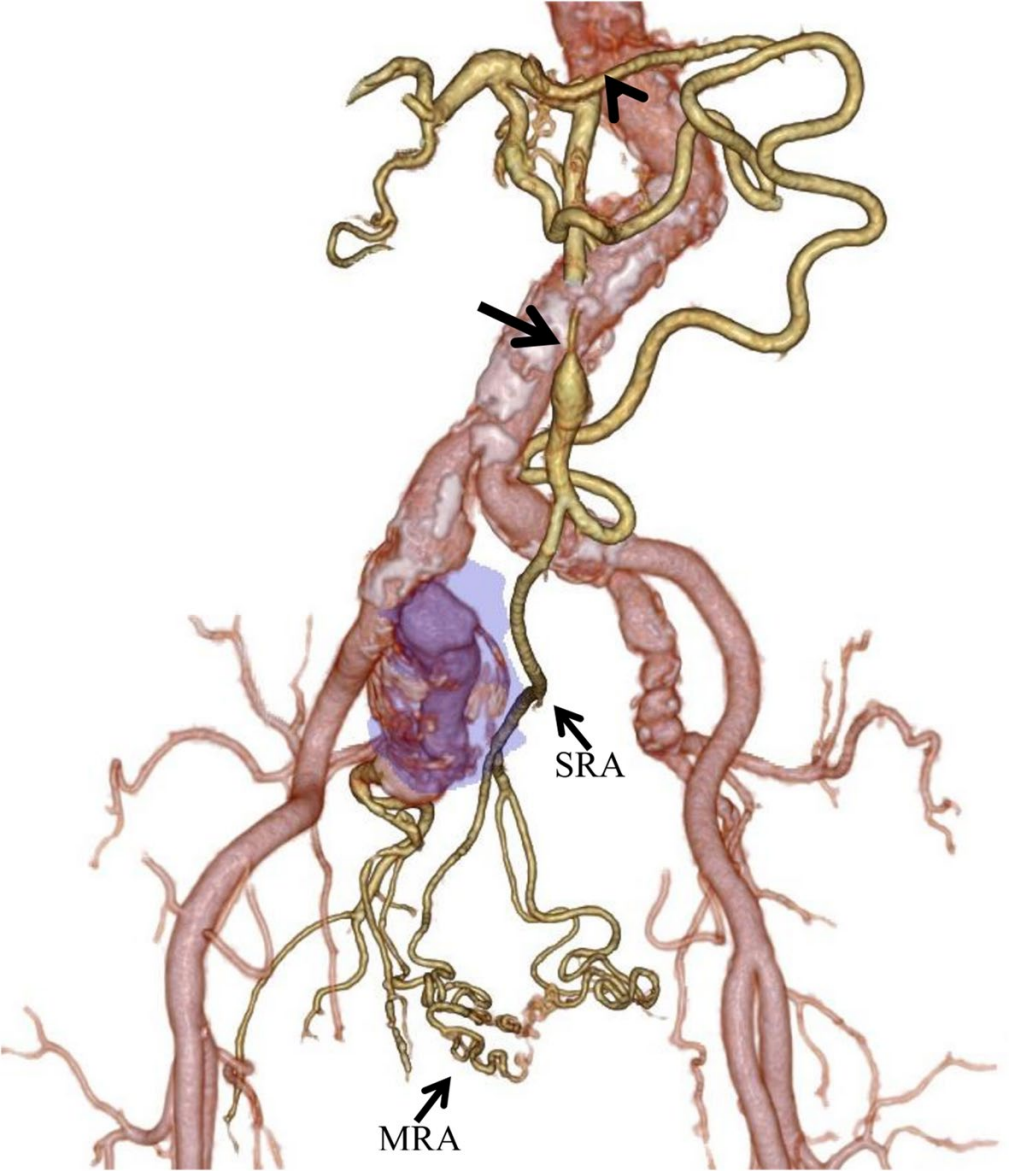
Three-dimensional reconstruction of preoperative contrast-enhanced CT. The celiacomesenteric trunk (arrowhead) is occluded and not visualized. The proximal part of the inferior mesenteric artery (thick arrow) is stenotic. The right middle rectal artery (MRA) and superior rectal artery (SRA) are dilated. The right internal iliac artery aneurysm is shown in purple

A 74-year-old male patient was monitored for bilateral common iliac artery (CIA) and right IIA aneurysms (Fig. 1). The patient was independent in daily activities and had a history of percutaneous coronary intervention for angina pectoris and paroxysmal atrial fibrillation. The IIA was gradually enlarged, exceeding the threshold for intervention and prompting surgical referral. Preoperative contrast-enhanced CT revealed right CIA and IIA aneurysms (maximal short-axis diameters of 34 and 48 mm, respectively), a severely stenotic proximal IMA (diameter < 1 mm), and an occluded CMT, likely due to median arcuate ligament compression, as indicated by its hooked appearance. Collateral pathways, including dilated left colic, accessory middle colic, and dorsal pancreatic arteries and the MRA, sustained liver and gastrointestinal blood flow (Fig. 2). The left MRA was visualized but was smaller compared with the right MRA, with no definite anastomosis to the superior rectal artery (SRA). Endovascular treatment was selected over open surgery owing to its minimally invasive nature and the patient’s preference for faster recovery. Embolization of the right IIA blocks the critical MRA collateral, thereby increasing the risk of hepatic and gastrointestinal ischemia. Thus, the occluded CMT was recanalized using a stent to restore antegrade blood flow through the CMT. Although preserving IIA flow using an iliac branch device was an option, this device was not used because the aneurysm extended distally into the IIA, owing to a severely restricted distal seal zone length of only 6 mm of healthy, non-aneurysmal, straight vessel segment in the IIA.

**Fig. 3:**
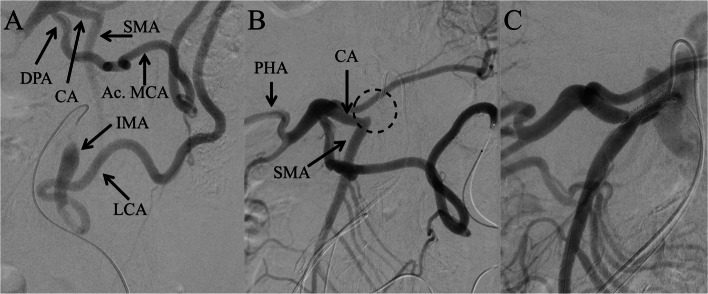
Angiographic images during recanalization of the occluded celiacomesenteric trunk. **A** Angiography of the inferior mesenteric artery. The left colic artery, accessory left colic artery, and dorsal pancreatic artery dilate as collateral vessels, and the celiac artery and superior mesenteric artery are enhanced. The superior rectal artery is not visualized, suggesting retrograde blood flow via the superior rectal artery. CA: celiac artery; SMA: superior mesenteric artery; DPA: dorsal pancreatic artery; Ac. MCA: accessory middle colic artery; IMA: inferior mesenteric artery; LCA: left colic artery. **B** Angiography of the left colic artery. The celiac artery, proper hepatic artery, and superior mesenteric artery are visible. The celiacomesenteric trunk (dotted circle) is not visualized because of occlusion. The origin of the celiacomesenteric trunk was determined using a shepherd hook catheter. **C** Angiography of the celiacomesenteric trunk after recanalization. The celiac, superior mesenteric, and peripheral arteries are shown

CMT recanalization was performed under local anesthesia. A 5-Fr guiding sheath (Parent Plus 45; Medikit, Japan) was inserted into the right common femoral artery, and a 5-Fr, 10-cm sheath into the left. Angiography of the IMA confirmed the collateral flow to the CA and SMA via the left colic, accessory middle colic, and dorsal pancreatic arteries (Fig. 3A). Angiography of the left colic artery, obtained using a 2.85-Fr microcatheter (Carry Leon High-Flow; UTM, Japan), visualized the proper hepatic artery and SMA (Fig. 3B). The CMT occlusion was successfully crossed antegradely using a 0.014-inch microguidewire (Astato XS 9–12; Asahi Intecc, Japan) and a 1.8-Fr microcatheter (Prominent Advance NEO; Tokai Medical Products, Japan). The microguidewire was exchanged for a 0.014-inch support wire (Aguru support; Boston Scientific, USA), followed by balloon dilatation with a 2.0 mm × 20 mm balloon catheter (Shiden, Kaneka Medix, Japan). A stent (Express SD 5 × 19 mm; Boston Scientific) was deployed, and angiography of the CMT confirmed restored flow to the common hepatic artery, splenic artery, and SMA (Fig. 3C).

After the procedure, the patient experienced abdominal pain without tenderness, raising concerns about intestinal ischemia. Contrast-enhanced CT showed small intestinal wall thickening and ascites but no poor enhancement of the small intestine with a patent CMT stent. The pain, likely due to reperfusion from the restored blood flow, resolved by day three, and the patient was discharged on day five. One month later, CT confirmed stent patency and normalization of the diameters of the MRA and SRA, indicating reduced collateral dependence.

**Fig. 4:**
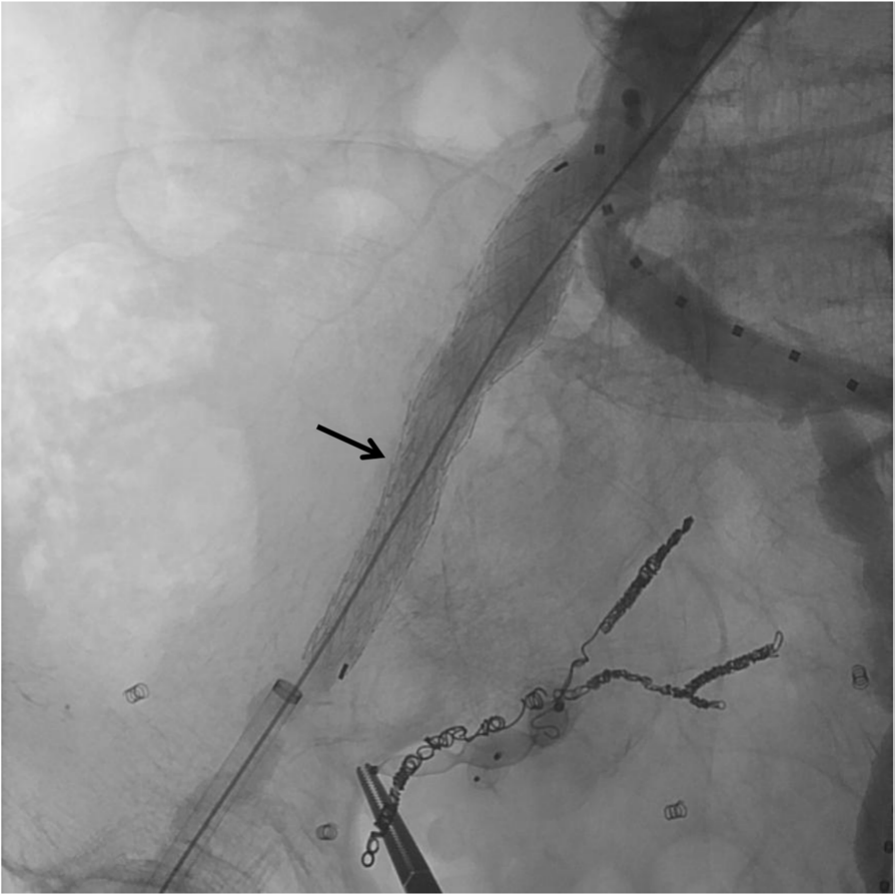
Left anterior oblique view of aortography after stent graft placement. A stent graft (arrows) is placed from the right common iliac artery to the right external iliac artery

A second procedure was performed 2 months after the initial procedure to allow sufficient time for collateral vessel remodeling and confirm sustained CMT stent patency, thereby minimizing the risk of severe ischemic complications. Under general anesthesia, a 9-Fr, 10-cm sheath was inserted into the right common femoral artery, and a 5-Fr guiding sheath (Parent Plus 45) into the left. Angiography verified the patency of the CMT. The right superior gluteal artery was embolized with a 10-mm Amplatzer Vascular Plug II (Abbott Laboratories, USA), the inferior gluteal artery with a 6-mm AVP IV, and the lateral sacral and obturator arteries with microcoils. The 9-Fr sheath was upsized to a 12-Fr sheath (Gore Dryseal Flex Introducer Sheath; W. L. Gore & Associates, Inc.), and a stent graft (Excluder Leg, 16 mm × 12 mm × 10 cm; W. L. Gore & Associates, Inc.) was deployed through the sheath from the right common artery to the external iliac artery (Fig. 4). The patient had been on dual antiplatelet therapy since coronary stenting. Following CMT stent placement, no additional antiplatelet or anticoagulant therapy was administered. Mild buttock claudication resolved within 1 month. The patient was discharged on day seven. At 1-year follow-up, no gastrointestinal ischemia, aneurysm growth (CT maximal short-axis diameter: CIA aneurysm, 33 mm; IIA aneurysm: 47 mm), or endoleaks were observed.

## Discussion

CMT occlusion, caused by atherosclerosis [4], thrombosis [5], or median arcuate ligament syndrome (MALS) [6], may lead to intestinal ischemia if untreated. Treatment options include bypass surgery, endovascular stenting, and ligament resection for MALS [6–8]. In this case, CMT occlusion and IMA stenosis increased the dependence on the MRA as collateral, making embolization risky. The dependence on the rectal splenic/colic axis for visceral perfusion mandated prior restoration of the normal CMT flow. CMT recanalization restores antegrade flow, thereby enabling the safe treatment of aneurysms. The greatest advantage of this approach is its low invasiveness. The disadvantage of this approach is that it is technically difficult to recanalize the occluded CMT, and if it cannot be recanalized, other methods are required. The IMA and IIA collaterals via the SRA and MRA are critical for aortoiliac occlusive disease [9]. As evidenced in this case, preoperative imaging, including CT and angiography, is essential for mapping collaterals and guiding treatment. In case of unsuccessful recanalization of the CMT, open surgery such as prosthetic graft replacement or bypass to the CMT could have been considered. Using an iliac branch device to preserve the IIA was also an option; several studies have demonstrated its efficacy and safety for IIA aneurysm repair [10, 11]. Gore Excluder Iliac Branch Endoprosthesis is the only iliac branch device available in Japan; neither this device nor a custom-made iliac branch device was used in this case owing to anatomical restrictions. Furthermore, transient postrecanalization pain, likely due to reperfusion, underscores the need for vigilant monitoring to distinguish between benign effects and true ischemia.

This case demonstrates that preoperative imaging, collateral mapping, and a staged endovascular approach with CMT recanalization followed by IIA stent graft placement can enable safe management of complex internal iliac artery aneurysms.

## Conclusion

The IIA branches may serve as vital collateral vessels in patients with CMT occlusion and IMA stenosis. In this case, we successfully employed a two-stage endovascular approach: CMT recanalization followed by embolization and stent graft placement. Thorough preoperative imaging and individualized treatment planning are crucial for managing complex vascular anatomies, ensuring optimal outcomes while minimizing ischemic complications.

## Data Availability

All data generated or analyzed during this study are included in this published article and its supplementary information files.
